# Post-earthquake trauma symptoms and their association with positive mental health among earthquake survivors in Türkiye

**DOI:** 10.1186/s40359-026-04855-1

**Published:** 2026-05-26

**Authors:** Deniz Yavuz Baskiran, Berna Bayir

**Affiliations:** 1 Inonu University Faculty of Medicine Liver Transplantation Institute, Malatya, Turkey; 2 KTO Karatay University School of Health Sciences Department of Nursing, Konya, Turkey

**Keywords:** Earthquake, Positive mental health, Trauma symptoms, Public health, Türkiye

## Abstract

**Background:**

Post-earthquake trauma symptoms are among the most common mental health outcomes following large-scale natural disasters. This study aimed to determine the level of post-earthquake trauma symptoms and examine their association with positive mental health among earthquake survivors in Türkiye.

**Method:**

This cross-sectional study was conducted in Malatya, one of the provinces most affected by the February 6, 2023 earthquakes, between May 30 and July 25, 2023. The sample consisted of 416 adults who experienced the earthquake. Data were collected using a Personal Information Form, the Scale for Determining the Level of Post-Earthquake Trauma, and the Positive Mental Health Scale. Data were analyzed using descriptive statistics, independent samples t-test, Pearson correlation analysis, and linear regression analysis.

**Results:**

Significant differences in post-earthquake trauma symptoms and positive mental health were observed according to demographic characteristics (*p* < 0.05). Women, individuals with higher education levels, and those who experienced property or financial loss reported higher levels of trauma symptoms. A statistically significant negative correlation was found between positive mental health and post-earthquake trauma symptoms (*r*= -0.413, *p* < 0.001). Linear regression analysis indicated that gender, property/financial loss, and positive mental health were significantly associated with trauma symptoms.

**Conclusions:**

The findings suggest that positive mental health is inversely associated with post-earthquake trauma symptoms and is associated with lower levels of in post-disaster psychological outcomes. Identifying vulnerable groups and strengthening positive mental health through community-based interventions may contribute to reducing the psychological impact of disasters. These results highlight the importance of integrating mental health promotion strategies into post-disaster public health planning.

## Background

Due to its tectonic location, Türkiye is one of the most earthquake‑prone countries in the world, and seismic events have historically caused extensive loss of life and property. The Eastern Anatolian Fault, which has generated destructive earthquakes in both historical and instrumental periods, remained relatively quiescent in terms of large earthquakes until the February 6, 2023 disaster. On that day, twin earthquakes with moment magnitudes of Mw 7.7 and Mw 7.6 struck southeastern Türkiye, leading to more than 50,000 deaths, over 115,000 injuries, and the collapse of tens of thousands of buildings [1, 2]. 

Earthquakes can have profound effects on mental health in both the immediate and prolonged aftermath of the disaster. Survivors often experience emotional trauma, disruption of social and family functioning, and a range of psychological problems, including post‑traumatic stress, depression, anxiety, sleep disturbances, and exacerbation of pre‑existing psychiatric conditions [3–5]. The prevalence of PTSD among survivors varies across contexts, with meta‑analyses suggesting that a substantial proportion of individuals exposed to major earthquakes meet criteria for probable PTSD in the months following the event, and PTSD frequently co‑occurs with depressive and anxiety symptoms [6]. While much of the disaster mental health literature has focused on psychopathology, there is a growing recognition of positive psychological outcomes and resources such as resilience, well‑being, and post‑traumatic growth. These constructs reflect adaptive psychological functioning and the capacity to maintain or regain mental health despite adversity [7]. In contrast, Borinca et al. (2025) demonstrated that traumatic memories influence reconciliation outcomes through metahumanization and dehumanization processes in a post-conflict context [8]. Among these, positive mental health has been conceptualized as a multidimensional construct encompassing emotional, psychological, and social well-being, rather than merely the absence of mental illness [9]. Importantly, contemporary models emphasize that mental health and mental illness are related but distinct dimensions, meaning that individuals may experience trauma-related symptoms while still maintaining aspects of positive mental health [10]. From a theoretical perspective, positive mental health may influence trauma-related outcomes through mechanisms such as emotional regulation, adaptive cognitive appraisal, and social functioning. Individuals with higher levels of positive mental health are more likely to interpret stressful experiences in less threatening ways and maintain better psychological functioning under stress [9, 11]. Recent research on survivors of the 2023 Kahramanmaraş earthquakes indicates that, several years post-disaster, many individuals not only experience ongoing trauma-related distress but also exhibit reduced levels of well-being and resilience, and that distress symptoms are strongly associated with lower well-being [12]. These findings highlight the importance of considering both negative and positive dimensions of mental health in post-disaster research. However, although previous studies have examined psychological distress and well-being following disasters, the role of positive mental health as a distinct construct in relation to trauma symptoms has received relatively limited attention, particularly in disaster-affected populations. From a theoretical perspective, positive mental health may influence trauma-related outcomes through mechanisms such as emotional regulation, cognitive appraisal, and interpersonal functioning. In this context, examining the association between positive mental health and post-earthquake trauma symptoms may provide additional insight into psychological functioning following large-scale disasters. Therefore, this study aimed to examine whether positive mental health is associated with post-earthquake trauma symptoms beyond demographic and disaster-related characteristics.

### Hypotheses

- H1: Post-earthquake trauma symptoms and positive mental health differ according to selected socio-demographic and disaster-related characteristics of earthquake survivors.
- H2: Positive mental health is negatively associated with post-earthquake trauma symptoms among earthquake survivors.

## Methods

### Design of the study

The study was conducted in Malatya province between May 30 and July 25, 2023, which suffered great losses in the February 6 earthquake in Türkiye. The design of the study is cross-sectional and correlational study from analytical research types.

### Population and sample of the study

The population of the study consisted of all adult individuals who experienced the earthquake in a family health center in Malatya. Participants were approached in a family health center during routine visits for various reasons, including general health check-ups and other healthcare services. Individuals who met the inclusion criteria and voluntarily agreed to participate were included in the study. Malatya, where the study was conducted, is located in close proximity to the epicenter of the February 6, 2023 Kahramanmaraş earthquakes and was among the provinces most severely affected in terms of structural damage and human loss. Therefore, the study population represents individuals with substantial exposure to the disaster. The sample size of the study was calculated with the G-Power 3.1.9.1 software. According to the Positive Mental Health Scale, the article by Filiztekin and Arabacı (2022) [13] was taken as a reference. Since the Scale for Determining the Level of Post-Earthquake Trauma was not used in any previous study, it could not be included in the calculation. According to the Positive Mental Health Scale, the minimum sample size was determined to be 330 with 95% power, 0.36 effect size and 0.05 margin of error. Since a clearly defined sampling frame was not available, a probabilistic sampling method could not be applied. The sample group of the study consisted of 416 participants who were recruited using a convenience sampling approach. Individuals who felt the earthquake tremors, were over the age of 18, could communicate, had an internet connection, could read and write, and could use at least one technological device were included in the study. Individuals who were in the active treatment period due to a physical injury suffered after the earthquake and individuals who were being treated for any mental health problem were not included in the study.

### Data collection process

Data were collected between May 30 and July 25, 2023, approximately three months after the February 6, 2023 earthquakes. The dependent variables of the study were post-earthquake trauma levels and positive mental health, while the independent variables included socio-demographic characteristics and unfavorable environmental conditions experienced after the earthquake. Data were collected using the Personal Information Form, the Post-Earthquake Trauma Scale, and the Positive Mental Health Scale. To ensure that only eligible participants were included, the Personal Information Form contained questions about current residence (limited to Malatya province) and direct exposure to the earthquake, such as being present in Malatya during the disaster, witnessing destruction, or experiencing personal or family loss. Only individuals who confirmed both their residence in Malatya and direct exposure to the earthquake were included in the study. All data were collected through face-to-face interviews, during which participants completed the questionnaires and scales themselves. The research team provided verbal explanations about the study’s purpose and voluntary participation and guided participants as needed. The completion time for the questionnaires was approximately 10–15 min. Although an online survey link was prepared to minimize paper usage, all participants provided their data through face-to-face interviews.

### Personal information form

In this form, which was created by the researchers by utilizing the literature [14, 15], there are 11 multiple-choice questions questioning the demographic characteristics of the participants such as age, gender, marital status, marital status, having children, education level, as well as their losses related to the earthquake.

### Scale for determining the level of post-earthquake trauma

This scale, developed by Tanhan and Kayri (2013) [16], was designed to assess the trauma levels of adults following an earthquake, and its validity and reliability have been established. The Likert-type scale includes a total of 20 items with options such as “strongly disagree”, “slightly agree”, “moderately agree”, “strongly agree” and “completely agree”. The scale has five sub-dimensions: Behavioral Problems (Items 1,2,3,4), Excitement Limitation (Items 5,6,7,8,9), Affective (Items 10,11,12,13), Cognitive Configuration (Items 14,15,16,17), and Sleep Problems (Items 18,19,20). Items 11 and 12 are reverse scored. It is stated that the scale does not have a cut-off score. The lowest score that can be obtained from the scale is 20 and the highest score is 100. The increase in the scores obtained from the scale indicates that the level of being affected by the earthquake increases. The internal consistency coefficient value of the original scale is 0.87 [16]. The Cronbach’s alpha internal consistency coefficient obtained in this study is 0.92. Although the Post-Earthquake Trauma Scale has demonstrated good validity and reliability in assessing trauma-related stress responses within the Turkish context, it remains a context-specific instrument and has not been extensively validated against widely used international measures such as the PTSD Checklist for DSM-5 (PCL-5).

### Positive mental health scale

The scale, which was adapted into Turkish by Teke and Arabacı (2018) [17], was developed by Lluch in 1999 [18]. The four-point Likert-type scale consists of 39 items in total. There are six sub-dimensions in total: Personal Satisfaction (Items 4, 6, 7, 12, 14, 31, 38, 39), Prosocial Attitude (Items 1, 3, 23, 25, 37), Self-Control (Items 2, 5, 21, 22, 26), Autonomy (Items 10, 13, 19, 33, 34), Problem solving and self-actualization (Items 15, 16, 17, 27, 28, 29, 32, 35, 36) and Interpersonal Relationship Skills (Items 8, 9, 11, 18, 20, 24, 30). Among these items, items 1, 2, 3, 6, 7, 8, 9, 10, 12, 13, 14, 19, 24, 30, 31, 33, 34, 38 and 39 are reverse coded. The item expressions are scored as “Always/almost” 4 points, “Often” 3 points, “Sometimes” 2 points, “Never/Rarely” 1 point. The highest score that can be obtained from the scale is 156 and the lowest score is 39. A high score on the scale indicates positive mental health. The internal consistency coefficient value of the original scale is 0.93 [17]. The Cronbach’s alpha internal consistency coefficient obtained from this study is 0.91.

### Study ethics

After the necessary screening for the research, ethical approval was obtained from XXX University Pharmaceutical and Non-Medical Device Research Ethics Committee (Decision date: 25/05/2023, Decision no: 2023/044). Permission was obtained from the authors who developed the scales used in the study or conducted validity and reliability studies. Individuals who met the inclusion criteria were informed about the purpose of the study, that participation in the study was voluntary, that they would not suffer any financial loss, and that the results would be used for scientific purposes. After this explanation, verbal and written informed consent was obtained from the participants who agreed to participate in the study. The conditions of the Declaration of Helsinki were complied with at every stage of the study.

### Statistical analysis of the data

Data were analyzed using SPPS 25 software (IBM Corp. Released 2017.IBM SPSS Statistics for Windows, Version 25.0.Armonk, NY: IBM Corp.). Descriptive statistics (mean, standard deviation, number and percentile) were given for categorical and continuous variables. In addition, the assumption of normality, one of the prerequisites of parametric tests, was examined with the “Shapiro-Wilk” test. To enhance statistical rigor, multiple assumption checks were performed. In addition to the Shapiro–Wilk test, homogeneity of variance was tested using Levene’s test, and graphical methods (Q–Q plots and histograms) were examined. Overall, the assumptions for parametric tests and regression analysis were met (*p* > 0.05). Differences between the means of independent variables were evaluated by Student t test. The relationship between two continuous variables was analyzed with Pearson Correlation Coefficient. Linear Regression analysis was used to determine the relationship between variables. In the regression analysis, multicollinearity diagnostics (VIF and tolerance) indicated no significant multicollinearity among predictors (VIF < 5). Independence of residuals was confirmed via the Durbin–Watson statistic. Although Likert-type scales are ordinal by design, total scores were analyzed as continuous variables using ordinary least squares regression, which is widely accepted when scales include multiple items and approximate interval-level measurement properties. Given the multiple statistical tests conducted, there is an increased risk of Type I error. While no formal multiple comparison correction was applied to preserve statistical power, this issue has been acknowledged in the limitations section, and results are interpreted with caution.

## Results

Table 1 shows the values of demographic variables and the changes that these variables caused on the averages. There were statistically significant differences in the levels of post-earthquake trauma and positive mental health according to the demographic characteristics of the participants such as age, gender, education, being trapped under rubble and experiencing loss of property/money (*p* < 0.05). According to age groups, it was determined that the positive mental health levels of the participants in the 36–45 age range were significantly lower than those in the 18–35 age group (Post Hoc *p* = 0.022), and the positive mental health levels of those who were under rubble were statistically significantly higher with a mean of 135.33 ± 2.51 (*p* < 0.05). When the post-earthquake trauma levels of the participants were evaluated, it was found that the mean post-earthquake trauma levels of women with 68.82 ± 16.88 and those who experienced loss of property or money with 64.51 ± 19.11 were significantly higher (*p* < 0.05). It was determined that those with an education level of university or higher had a significantly higher (post hoc: 0.004) mean post-earthquake trauma level with a mean of 65.65 ± 17.25 compared to primary school graduates (53.53 ± 20.70) (Table 1).

**Table 1:** Comparison of demographic variables according to level of post-earthquake trauma and level of positive mental health

Demographic Variables		Frequencies		Post Earthquake Trauma	*t/F/p*	Positive Mental Health	*t/F/p*
		*N*	*%*	*‾X ± SD*		*‾X ± SD*	
Age (yaear)^¥^	18–35	194	46.6	62.45 ± 19.58	0.219/0.804	120.91 ± 19.30	3.573/**0.029**
	36–45	159	38.2	61.35 ± 18.95		115.41 ± 18.41	***PH p*** **= 0.022**
	≥ 46	63	15.1	62.98 ± 18.99		117.93 ± 21.32	
Gender^ʈ^	Female	194	46.6	68.82 ± 16.88	7.042/**0.000**	118.04 ± 17.24	-0.311/0.756
	Male	222	53.4	56.24 ± 19.25		118.63 ± 21.15	
Marital Status^ʈ^	Married	296	71.2	62.35 ± 18.87	0.392/0.696	117.86 ± 19.50	-0.833/0.406
	Single	120	28.8	61.51 ± 20.12		119.60 ± 19.19	
Having a child^ʈ^	Yes	273	65.6	62.52 ± 18.79	0.584/0.559	118.04 ± 19.36	-0.467/0.641
	No	143	34.4	61.33 ± 20.05		118.97 ± 19.54	
Education^¥^	Primary school	30	7.2	53.53 ± 20.70	7.332/**0.001**	116.03 ± 19.60	0.291/0.748
	High school	203	48.8	60.18 ± 20.08	***PH p*** **= 0.004**	118.22 ± 20.62	
	≥ University	183	44.0	65.65 ± 17.25		118.90 ± 18.02	
Location in the earthquake^¥^	In a building	385	92.5	61.92 ± 19.20	1.144/0.319	118.58 ± 19.29	0.604/0.547
	In a workplace	19	4.6	68.10 ± 17.12		113.57 ± 20.75	
	In an open area	12	2.9	58.58 ± 22.60		118.66 ± 21.66	
Under rubble^ʈ^	Yes	3	0.7	44.33 ± 20.25	-1.526/0.265	135.33 ± 2.51	9.828/**0.001**
	No	413	99.3	62.24 ± 19.18		118.23 ± 19.42	
Death of a relative^ʈ^	Yes	95	22.8	61.75 ± 19.19	-0.205/0.838	119.15 ± 18.10	0.477/0.634
	No	321	77.2	62.21 ± 19.25		118.12 ± 19.80	
Loss of property/ money^ʈ^	Yes	240	57.7	64.51 ± 19.11	3.006/**0.003**	117.92 ± 18.89	-0.537/0.592
	No	176	42.3	58.84 ± 18.93		118.96 ± 20.12	

^¥^ One way ANOVA (*F* value is given for trivariate analysis), ^ʈ^ Independent samples *t* test (*t* value is given for bivariate analysis). *X ± SD*: Mean±Standard Deviation, *PH*: Post Hoc analysis

Bold indicates *p*<0.05

Table 2 shows the correlation between the participants’ post-earthquake trauma levels and their positive mental health levels. When the relationship between the sub-dimensions and total scores of the scales used was evaluated, it was determined that there was a statistically significant, negative, weak correlation between the sub-dimensions of Personal Satisfaction, Prosocial Attitude, Self-Control, Autonomy, Interpersonal Relationship Skills, which are sub-dimensions of the Positive Mental Health Level Scale, and all sub-dimensions of the Post-Earthquake Trauma Level Determination Scale (*p* < 0.001). There was a very weak negative correlation between Problem Solving and Self-Actualization sub-dimensions of the Positive Mental Health Level Scale and all sub-dimensions of the Post-Earthquake Trauma Level Assessment Scale except Behavioral Problems and Cognitive Structuring sub-dimensions. When a comparison was made according to the total scores of the scales, it was determined that there was a statistically significant negative moderate correlation (r:-0.413, p:0.000) between the total score of the Positive Mental Health Level Scale and the total score of the Post-Earthquake Trauma Level Assessment Scale (Table 2).

**Table 2:** Comparison of the relationship between participants’ post-earthquake trauma levels and positive mental health levels

Sub-Dimensions	1	2	3	4	5	PET Total Score
Personal Satisfaction	-0.354**	-0.507**	-0.335**	-0.352**	-0.370**	-0.480**
	0.000	0.000	0.000	0.000	0.000	0.000
Prosocial Attitude	-0.174**	-0.265**	-0.228**	-0.164**	-0.231**	-0.260**
	0.000	0.000	0.000	0.001	0.000	0.000
Self-Audit	-0.258**	-0.350**	-0.274**	-0.269**	-0.256**	-0.348**
	0.000	0.000	0.000	0.000	0.000	0.000
Autonomy	-0.224**	-0.357**	-0.194**	-0.255**	-0.251**	-0.325**
	0.000	0.000	0.000	0.000	0.000	0.000
Problem Solving and Self-Actualisation	-0.068	-0.193**	-0.255**	-0.081	-0.098*	-0.163**
	0.163	0.000	0.000	0.100	0.045	0.001
Interpersonal Relationship Skills	-0.168**	-0.275**	-0.203**	-0.179**	-0.173**	-0.248**
	0.001	0.000	0.000	0.000	0.000	0.000
PMHS Total Score	-0.279**	-0.443**	-0.345**	-0.292**	-0.308**	-0.413**
	0.000	0.000	0.000	0.000	0.000	0.000

*p < 0.05, **p < 0.001, Pearson Correlation Coefficient (*r*); Summary statistics are given as r (*p*) value. 1: Behavioural Problems, 2: Excitement Limitation, 3: Affective, 4: Cognitive Configuration, 5: Sleep Problems. *PET* Post-Earthquake Trauma, Level Determination Scale, PMHS: Positive Mental Health Scale

The effect of some demographic variables and Positive Mental Health Scale levels of the participants on the mean scores of the Scale for Determining the Level of Post-Earthquake Trauma was determined by Linear Regression analysis. According to the results of the analysis, gender, loss of property or money, and Positive Mental Health Scale level were found to be significantly associated with post-earthquake trauma scores, whereas age, education, marital status, having a child, location during the earthquake, being under rubble, and death of a relative were not statistically significant in the model. In line with the findings, being male was associated with a decrease of 11.530 points in the mean score of the Scale for Determining the Level of Post-Earthquake Trauma (*t*: -6.632, *p*: 0.000), and not experiencing property or financial loss was associated with a decrease of 6.497 points (*t*: -3.973, *p*: 0.000). It was also found that higher Positive Mental Health Scale scores were associated with lower post-earthquake trauma scores (*B*: -0.399, *t*: -9.665, *p*: 0.000). The overall model was statistically significant (F: 16.942, *p*: 0.000) and explained approximately 31% of the variance in post-earthquake trauma scores (*R²* = 0.316). Multicollinearity diagnostics indicated no significant multicollinearity among predictors (VIF range: 1.01–2.59). The overall model was statistically significant (F = 16.942, *p* < 0.001) and explained approximately 31.6% of the variance in post-earthquake trauma scores, indicating moderate explanatory power. Among the predictors, positive mental health emerged as the strongest factor (β = -0.403, *p* < 0.001), followed by gender and property/financial loss (Table 3).

**Table 3:** Comparison of the effect of some demographic variables and positive mental health levels of the participants on post-earthquake trauma levels by regression analysis

Variables		Unstandardised		Standardised			*CI*		
		*B*	*SH*	*Beta*	*t*	*p*	Lower Bound	Upper Bound	VIF
Scale for Determining the Level of Post-Earthquake Trauma	Constant	132.399	23.039	-	5.747	0.000**	87.108	177.690	
	Age	-0.179	0.109	-0.085	-1.640	0.102	-0.393	0.036	1.569
	Gender	-11.530	1.739	-0.300	-6.632	0.000**	-14.948	-8.113	1.203
	Education	2.665	1.449	0.085	1.840	0.067	-0.183	5.514	1.247
	Marital status	-1.993	2.675	-0.047	-0.745	0.457	-7.252	3.266	2.352
	Having a child’s	-1.232	2.684	-0.030	-0.459	0.646	-6.507	4.043	2.599
	Location in the earthquake	0.855	2.054	0.017	0.417	0.677	-3.182	4.892	1.015
	Under rubble	8.172	9.461	0.036	0.864	0.388	-10.427	26.772	1.026
	Death of a relative	0.658	1.912	0.014	0.344	0.731	-3.101	4.418	1.031
	Loss of property/money	-6.497	1.635	-0.167	-3.973	0.000**	-9.712	-3.283	1.043
	PMHSTotal Score	-0.399	0.041	-0.403	-9.665	0.000**	-0.480	-0.318	1.020
	Durbin-Watson= 1.909 *R*= 0.562^a^*R*^2^= 0.316 Adj *R*^2^= 0.297 F= 16.942 *p*= 0.000^b**^								

*SH* Standard Error, *CI* Confidence Interval, *PMHS* Positive Mental Health Scale, *VIF* Variance Inflation Factor. All VIF values were below 5

**p<0,01 1: Regression Analysis (F); 2: Coefficient Analysis (t); Summary statistics are given as Regression Coefficient (Standard Error) value

## Discussion

Natural disasters such as earthquakes can have wide-ranging negative effects on individuals, encompassing psychological, physical, social, and economic domains. Disaster survivors often experience mental health problems, including trauma symptoms, depression, and anxiety [19–21]. The present study aimed to assess post-earthquake trauma and positive mental health among survivors of the February 6, 2023 earthquakes, addressing three primary research questions. First, the study examined differences in post-earthquake trauma and positive mental health according to demographic variables among residents of Malatya. The results indicated that the middle-aged group (36–45 years) reported lower positive mental health, while those rescued from under the rubble showed higher positive mental health. In this study, the unexpectedly higher positive mental health of individuals rescued from under rubble was observed. However, the number of participants in this subgroup was very small (*n* = 3), and therefore this finding should be interpreted with caution and cannot support meaningful conclusions. In addition, no statistically significant differences in post-earthquake trauma levels were observed according to age, marital status, having children, location during the earthquake, being under rubble, and loss of a relative. This finding differs from some previous studies, which have identified exposure-related experiences such as being trapped under rubble, fear, injury, or loss as significant predictors of post-traumatic stress disorder [22]. This discrepancy may be related to differences in sample characteristics, timing of data collection, or measurement approaches across studies. This situation highlights the importance of individual characteristics, experiences, and circumstances during the periods before, during, and after an earthquake [23]. Furthermore, given the cross-sectional design of the study, the relationship between positive mental health and trauma symptoms should be interpreted as potentially bidirectional, as higher trauma levels may also negatively influence positive mental health.

The analysis of post-earthquake trauma levels revealed that individuals with higher education reported greater trauma. The analysis of post-earthquake trauma levels revealed that individuals with higher education reported greater trauma. This finding should be interpreted with caution, as the underlying mechanisms were not directly examined in this study and no causal explanation can be established. This result differs from some previous studies that have associated lower educational levels with increased vulnerability to trauma-related symptoms [22, 24]. This unexpected finding may be explained by differences in psychological awareness and reporting tendencies, as individuals with higher educational levels may be more attuned to emotional and cognitive symptoms of distress. Moreover, higher levels of education may be associated with more complex cognitive appraisal processes, potentially leading to heightened perception and reporting of trauma-related symptoms. However, this result may also reflect contextual or sample-specific factors and warrants further investigation. Therefore, this finding may reflect sample-specific or contextual factors and should be interpreted cautiously.

Trauma levels were also higher among those who experienced property or financial loss but were not trapped under rubble. These results align with previous research showing that fear, exposure to material and life losses, gender (e.g., being female), prior trauma, traumatic distress, lack of social support, age extremes, and low educational level are associated with PTSD, depression, and anxiety symptoms [22, 24–26]. 

Another key finding was the negative relationship between post-earthquake trauma and positive mental health. In addition to the overall association between positive mental health and post-earthquake trauma, the analysis of subdimensions revealed that several components of positive mental health, particularly personal satisfaction, self-control, and autonomy, showed relatively stronger negative correlations with trauma-related symptoms. This suggests that specific aspects of positive mental health may be more closely linked to certain dimensions of trauma. As participants’ psychological well-being decreased, their trauma levels increased. This aligns with evidence that natural disasters affect mental health and that secondary stressors, such as separation from family, loss of pets, or disruption of daily routines, can exacerbate psychological distress [27, 28]. Studies with child and adolescent populations suggest that acceptance, emotional expression, and cognitive reframing enhance resilience and mitigate disaster-related psychological impacts [29–31]. Similarly, in this study, higher positive mental health was associated with better coping, and lower levels of post-earthquake trauma symptoms were observed. The findings of this study should also be interpreted within the broader cultural context of Türkiye, where resilience is often embedded in collective and religious frameworks rather than purely individual psychological processes. Social cohesion, family interconnectedness, and culturally shared meanings of suffering may influence how individuals interpret and cope with trauma. In particular, religious coping—such as prayer, spiritual acceptance, and belief in fate—may function as a protective factor by facilitating cognitive reframing and emotional regulation. These culturally grounded coping strategies may interact with positive mental health constructs and influence trauma-related outcomes. However, as these variables were not directly assessed, their potential contribution remains speculative and warrants further investigation. Due to the cross-sectional nature of the study, the findings reflect associations rather than causal or predictive relationships, and reverse causality cannot be ruled out.

Regression analyses indicated that gender, material/property loss, and positive mental health were significantly associated with trauma levels. Being female increased trauma scores by 11.614 points, while property/money loss increased scores by 6.226 points; higher positive mental health was associated with lower trauma scores. These findings are consistent with prior studies reporting elevated PTSD risk among women [32], and highlighting gender differences in maladaptive behaviors post-disaster [33]. These findings are consistent with previous studies reporting higher trauma-related symptoms among women following disasters [22, 24–26]. However, the underlying mechanisms of this difference were not directly examined in the present study. Material/property loss also emerged as a significant predictor, corroborating findings from the Wenchuan Earthquake study, although temporal proximity to the disaster in the current study may have influenced results [34]. Additional exposure-related and demographic variables were included in the regression model; however, these variables were not statistically significant, and the main findings remained consistent.

Finally, positive mental health was associated with lower levels of post-earthquake trauma symptoms. Individuals with greater psychological well-being are better able to cope with adverse events, preserving their mental and physical health [35]. Resilience, encompassing personal traits and social resources, facilitates recovery after traumatic events [36, 37], and is closely linked to positive mental health [38]. Prior research emphasizes that positive psychological well-being mitigates trauma-related mental health impacts [39, 40], supporting the current study’s findings that higher positive mental health is associated with lower levels of post-earthquake trauma symptoms. In summary, the study demonstrates that demographic factors (gender, education, and material loss) and positive mental health are associated with post-earthquake trauma levels. The reliance on a convenience-based sample may have introduced selection bias, as individuals with limited access to digital resources or lower functional capacity may be underrepresented. Therefore, the findings should be interpreted with caution in terms of generalizability to the broader population of earthquake survivors. Women, individuals with higher education, and those experiencing property/financial losses exhibited higher trauma, whereas positive mental health was associated with lower trauma levels. These findings underscore the importance of targeted interventions focusing on resilience and mental well-being to reduce disaster-related trauma. These findings may also be interpreted within the framework of Post-Traumatic Growth (PTG), which suggests that individuals can experience positive psychological changes alongside distress following traumatic events [41]. From this perspective, the coexistence of trauma symptoms and positive mental health observed in this study may reflect adaptive psychological processes rather than mutually exclusive outcomes.

### Limitation

Several limitations of the current study should be acknowledged. First, participants were recruited as self-identified earthquake survivors. Although all participants reported living in Malatya at the time of the study, the study did not include independent verification of their residency or direct confirmation of their experiences during the earthquake. The current sample was predominantly young, which may limit the generalizability of the findings, as younger individuals may have lower vulnerability and higher likelihood of accessing mental health support. Additionally, the study was conducted only two months after the earthquake, which may not have provided sufficient time for post-earthquake trauma symptoms to fully manifest in some participants. Consequently, long-term effects could not be observed. Data were collected through self-report measures, which are subject to social desirability bias, recall bias, and other reporting inaccuracies. The cultural context in which participants live may also influence the way they express emotions and seek help, potentially affecting the results. In addition, individuals receiving treatment for mental health problems were excluded, as ongoing treatment (e.g., pharmacological interventions) may influence the reporting of psychological symptoms and positive mental health. However, this criterion may have introduced selection bias by underrepresenting individuals with higher levels of distress. The study design was cross-sectional, limiting the ability to draw causal inferences and track changes over time. Finally, the findings may not be generalizable to all earthquake survivors, as the sample consisted solely of individuals from a specific region and/or those who applied to an aid organization. Future research should consider longitudinal designs, verified participant experiences, and formal mediation analyses to address these limitations and enhance the robustness of conclusions. Additionally, the very small number of participants in some subgroups (e.g., individuals rescued from under rubble) limits the reliability of these comparisons and prevents meaningful interpretation. Furthermore, the Post-Earthquake Trauma Scale, although validated in the Turkish context, may have limited comparability with internationally established instruments (e.g., PCL-5), which may affect cross-study comparisons. In addition, the absence of baseline mental health assessment prior to the earthquake limits the ability to distinguish between pre-existing psychological conditions and trauma responses specifically related to the disaster.

## Conclusions

This study was conducted to examine the trauma levels of individuals living in Malatya province following the earthquakes of 6 February 2023 and the effect of positive mental health on these levels. The findings revealed that women, those with higher levels of education, and individuals who experienced loss of property or money had significantly higher levels of trauma symptoms. Higher levels of positive mental health were significantly associated with lower levels of post-earthquake trauma symptoms. While the present study does not examine interventions directly, the findings may offer preliminary insights for post-disaster mental health strategies and highlight the potential relevance of strengthening positive mental health. Additionally, gender, material loss, and positive mental health significantly predicted post-earthquake trauma levels. The unexpectedly high levels of positive mental health among individuals rescued from the rubble should be interpreted with caution due to the very small subgroup size. Overall, the findings suggest that the effects of large-scale disasters such as earthquakes on individuals’ mental health are multidimensional, and that positive mental health is associated with lower levels of trauma symptoms. Based on these findings, it may be beneficial to consider prioritizing support for vulnerable groups, particularly women, individuals who have experienced material loss, and those with lower levels of positive mental health, in post-earthquake mental health services. Interventions such as psychoeducation, resilience-building group work, and social support programmes aimed at strengthening positive mental health may be considered as potentially relevant approaches to support mental well-being. In addition, early mental health screening and support for at-risk individuals may be considered, although the present study does not directly evaluate intervention effectiveness. Providing information about post-disaster psychological reactions and coping mechanisms may also be helpful in reducing potential post-traumatic stigma. Finally, strengthening psychosocial support services within a sustainable and community-based disaster management framework may be considered as a potential direction for future practice and research.

## Data Availability

The anonymized SPSS dataset used in this study, supporting the findings reported in this article, is publicly available at Zenodo.10.5281/zenodo.19220515
